# Synthesis, crystal structure, and Hirshfeld surface analysis of bis­{2-[(*E*)-(*p*-tolyl­imino)­meth­yl]benzen-1-olato}palladium

**DOI:** 10.1107/S2056989025011570

**Published:** 2026-01-06

**Authors:** Nur Nabihah Muzammil, Siti Syaida Sirat, Mohd Mustaqim Rosli, Muhamad Azwan Hamali, Mohd Tajudin Mohd Ali, Amalina Mohd Tajuddin

**Affiliations:** a Faculty of Applied Sciences, Universiti Teknologi MARA, Shah Alam, 40450 Shah, Alam, Selangor, Malaysia; b Atta-ur-Rahman Institute for Natural Product Discovery (AuRIns), UiTM Puncak, Alam, 42300, Bandar Puncak Alam, Selangor, Malaysia; c X-ray Crystallography Unit, School of Physics, Universiti Sains Malaysia, 11800, USM, Penang, Malaysia; Universidad de la República, Uruguay

**Keywords:** crystal structure, Schiff base, Pd^II^ complex, Hirshfeld surface analysis

## Abstract

The title compound, [Pd(C_14_H_12_NO)_2_], contains an *N,O*-bidentate ligand and features a square-planar Pd^II^ atom coordinated to two chelating ligands. Each ligand binds through one nitro­gen atom and one oxygen donor atom, forming two six-membered chelate rings. The Pd^II^ atom lies essentially within the coordination plane, with the *trans* arrangement of the donor atoms giving rise to the square-planar geometry. In the crystal, the mol­ecules are linked through weak C—H⋯π inter­actions, which direct the mol­ecular packing. To gain further insights into the inter­molecular contacts, a Hirshfeld surface analysis was performed.

## Abstract 2

The title compound, [C_28_H_24_N_2_O_2_Pd], contains an *N,O*-bidentate ligand and features a square-planar Pd^II^ atom coordinated to two chelating ligands. In the crystal, the mol­ecules are linked through weak C—H⋯π inter­actions.

## 1. Chemical context

The coordination chemistry of palladium(II) Schiff base complexes has been explored extensively due to their versatile structural motifs and wide ranging applications in catalysis, bioinorganic chemistry, and materials science (Kargar *et al.*, 2021 ▸). Schiff bases derived from aromatic aldehydes and amines, particularly those incorporating salicyl­aldehyde, have attracted significant attention in structural studies (Aggoun *et al.*, 2020 ▸). Among these, ligands formed by the condensation of salicyl­aldehyde with substituted anilines are known to stabilize square-planar Pd^II^ atoms while enabling systematic tuning of electronic and steric properties (El-Qisairi *et al.*, 2023 ▸). Numerous Pd^II–^Schiff base complexes bearing *N,O*-bidentate chelating ligands have been synthesized and structurally characterized, highlighting variations in Pd—N and Pd—O bond lengths reflecting the influence of ligand substituents (Celedón *et al.*, 2020 ▸; El-Qisairi *et al.*, 2023 ▸; Khanmoradi *et al.*, 2017 ▸).

Reports of palladium(II) complexes with salicyl­idene-*para*-toluidine derivatives are less frequent than those with unsubstituted salicylideneanilines. The incorporation of a *para*-methyl substituent into the aniline fragment can modify both the steric and electronic environments around the metal center, thereby influencing inter­molecular inter­actions and supra­molecular assembly in the solid state (Tudu *et al.*, 2024 ▸).

Herein, we report the synthesis of a Schiff base ligand obtained by the condensation of salicyl­aldehyde with *para*-toluidine, and its coordination to palladium(II) to yield a square-planar complex, C_28_H_24_N_2_O_2_Pd, **1**. The Schiff base ligand has been reported previously; however, complex **1** described in this work is new. It was characterized using solid-state analysis such as melting point, elemental analysis and IR spectroscopy, as presented in the experimental section. The single crystals suitable for X-ray diffraction were grown from the filtrate of the crude product; however, the amount obtained was insufficient for elemental analysis. Nevertheless, the pure crystalline product is expected to have the same elemental (C, H, N) composition as the analyzed crude sample, with only minor deviations (Tsionou *et al.*, 2017 ▸). Single-crystal X-ray diffraction and Hirshfeld surface analysis were employed to elucidate the influence of the *para*-methyl substituent on the structural parameters and inter­molecular inter­actions in this class of palladium(II) Schiff base derivatives.

In addition to the solid-state analyses, **1** was also fully characterized in solution using NMR and UV-Vis (see supporting information). These techniques provide additional evidence for successful complex formation and, importantly, indicate that the mol­ecular structure observed in the solid state is largely preserved in solution. The ^1^H and ^13^C NMR spectra showed the expected ligand coordination shifts, confirming that no structural rearrangement occurs upon dissolution in CDCl_3_. Furthermore, the solubility of **1** in this non-polar solvent is consistent with the Hirshfeld surface analysis, which revealed relatively weak inter­molecular inter­actions in the crystal packing (Hangan *et al.*, 2023 ▸). This correlation between solid-state inter­actions and solution behavior enhances our understanding of the structural stability of **1**.
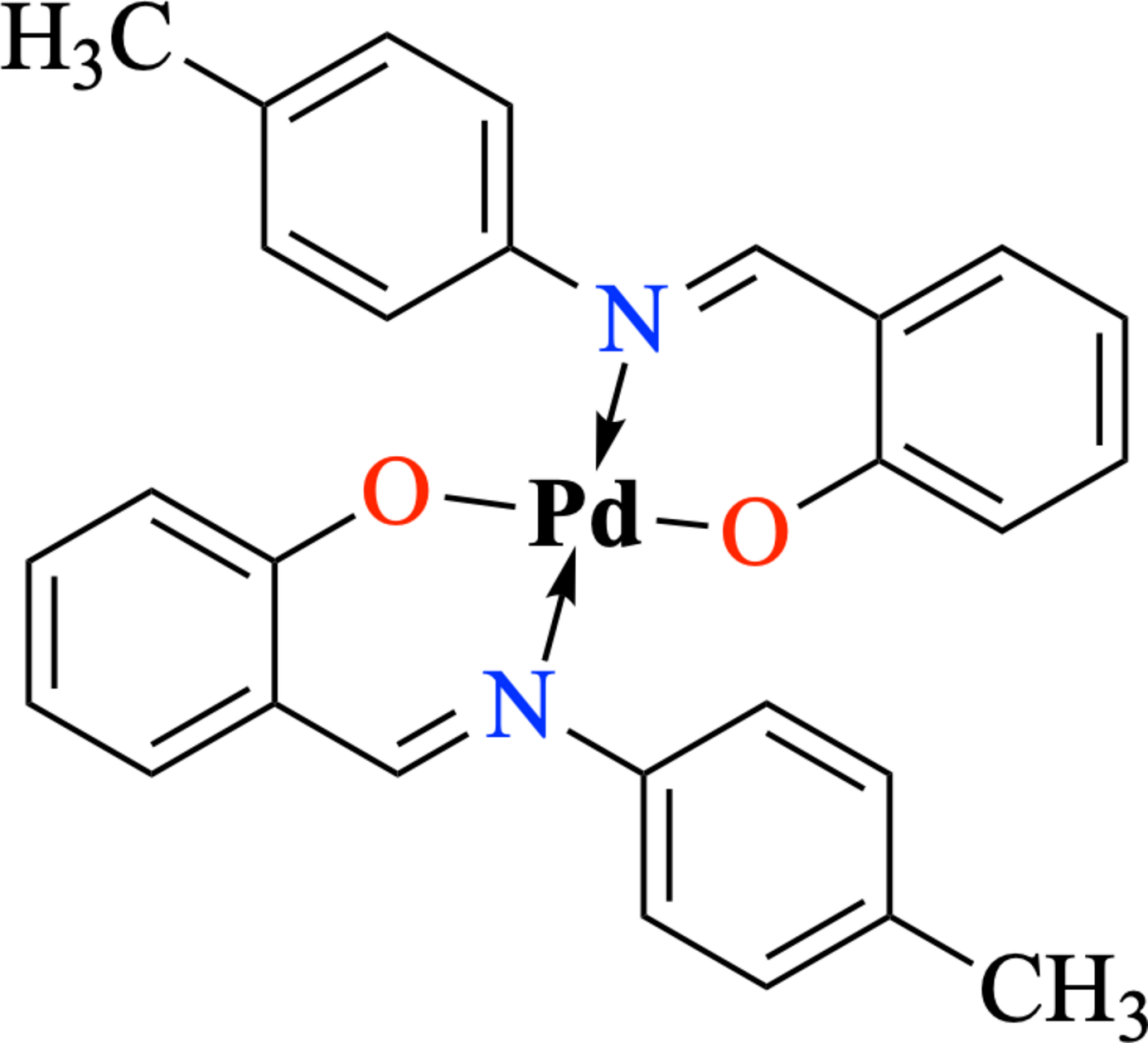


## 2. Structural commentary

**Figure 1:**
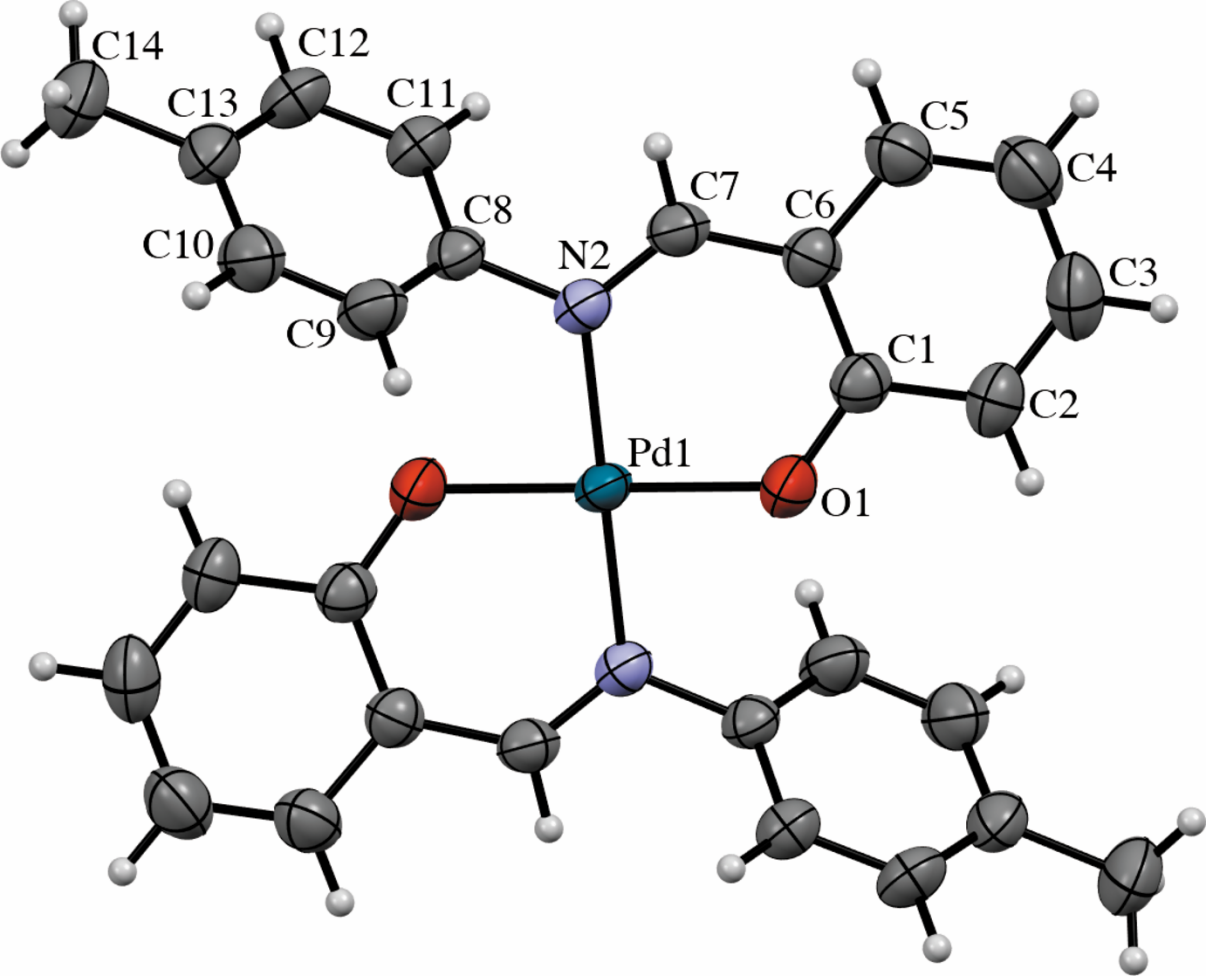
The mol­ecular structure of **1**, showing 50% probability displacement ellipsoids and the atom-numbering scheme. Unlabelled atoms are generated by the symmetry operation (−*x*, −*y*, −*z*).

The mol­ecular structure of **1** is shown in Fig. 1 ▸. It shows a crystallographically imposed centre of symmetry, with the Pd^II^ atom lying on an inversion center. The Pd^II^ atom adopts a square-planar geometry, being chelated by two *N,O*-bidentate ligands, C_14_H_13_NO, each chelating through one nitro­gen atom and one oxygen donor atom. The two benzene rings, C1–C6 and C8–C13, are planar with maximum deviations of 0.009 (5) and 0.008 (3) for atoms C5 and C8, respectively, from their mean square planes. The dihedral angle between the rings is 52.0 (2)°. All observed bond lengths and angles involving Pd and the ligand (Table 1 ▸) fall within the values expected from compounds previously reported by our team (Rosnizam *et al.*, 2022 ▸; Ahmad *et al.*, 2020 ▸; Mohd Tajuddin *et al.*, 2015 ▸).

## 3. Supra­molecular features

**Figure 2:**
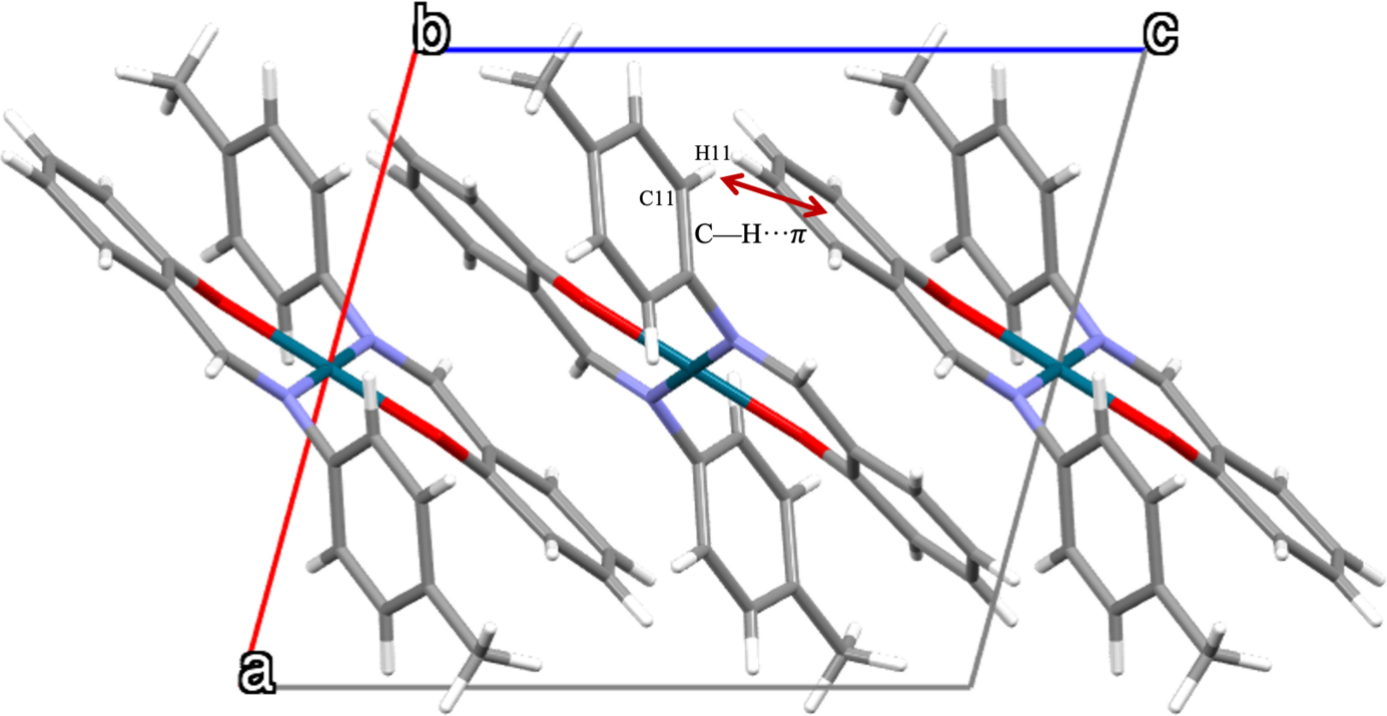
The crystal packing of **1**, viewed along *b*-axis.

In the crystal, the mol­ecules pack in a three-dimensional arrangement without the formation of inter­molecular hydrogen bonds or π–π stacking inter­actions. Adjacent mol­ecules are not parallel and adopt different orientations within the crystal structure. Fig. 2 ▸ shows the mol­ecular packing viewed along the *b*-axis direction. The only inter­action between mol­ecules is a weak C—H⋯π contact between C11—H11 and the centroid of the C1–C6 ring *Cg*3 (Table 2 ▸).

There is a short contact between Pd1 and H5 in the crystal structure, which is appeared in the Hirshfeld surface fingerprint plots. However, H5 is a hydrogen atom attached to carbon, positioned geometrically rather than found in the difference-Fourier map. Therefore, this contact should not be inter­preted as a significant Pd⋯H inter­action.

## 4. Hirshfeld surface analysis

**Figure 3:**
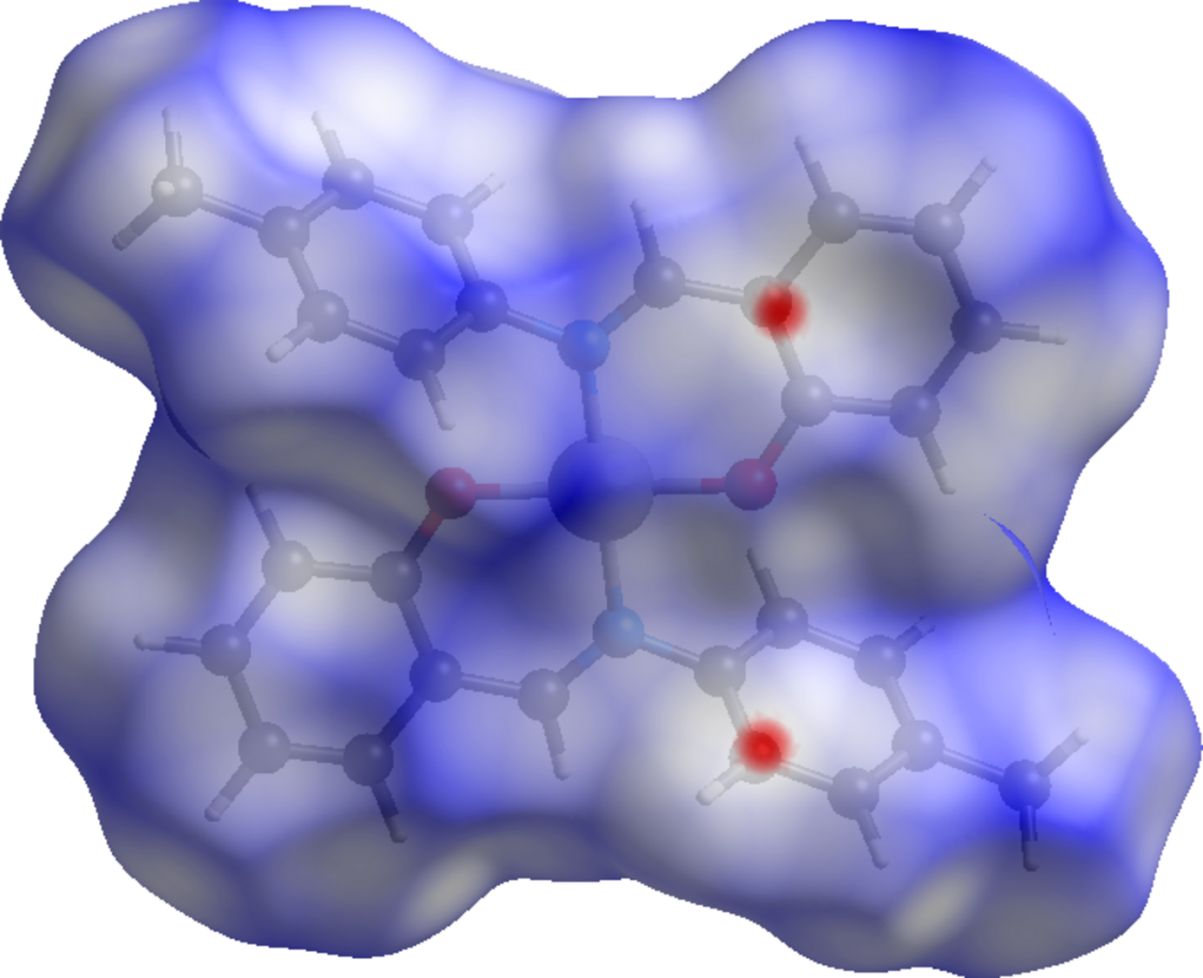
Hirshfeld surface of **1**, mapped over *d*_norm_.

A Hirshfeld surface analysis was carried out to investigate and visualize the inter­molecular inter­actions present between mol­ecules and, importantly, to qu­antify the individual contributions of these contacts to the overall packing (Gannouni *et al.*, 2023 ▸). The Hirshfeld surface was generated using *CrystalExplorer 21.5* (Fig. 3 ▸). Consistent with the crystallographic analysis, no strong hydrogen-bond inter­actions are observed in **1**. Instead, the Hirshfeld surface mapped over the *d*_norm_ displays several small bright-red spots, corresponding to weak and longer range inter­actions that contribute to the consolidation of the packing.

**Figure 4:**
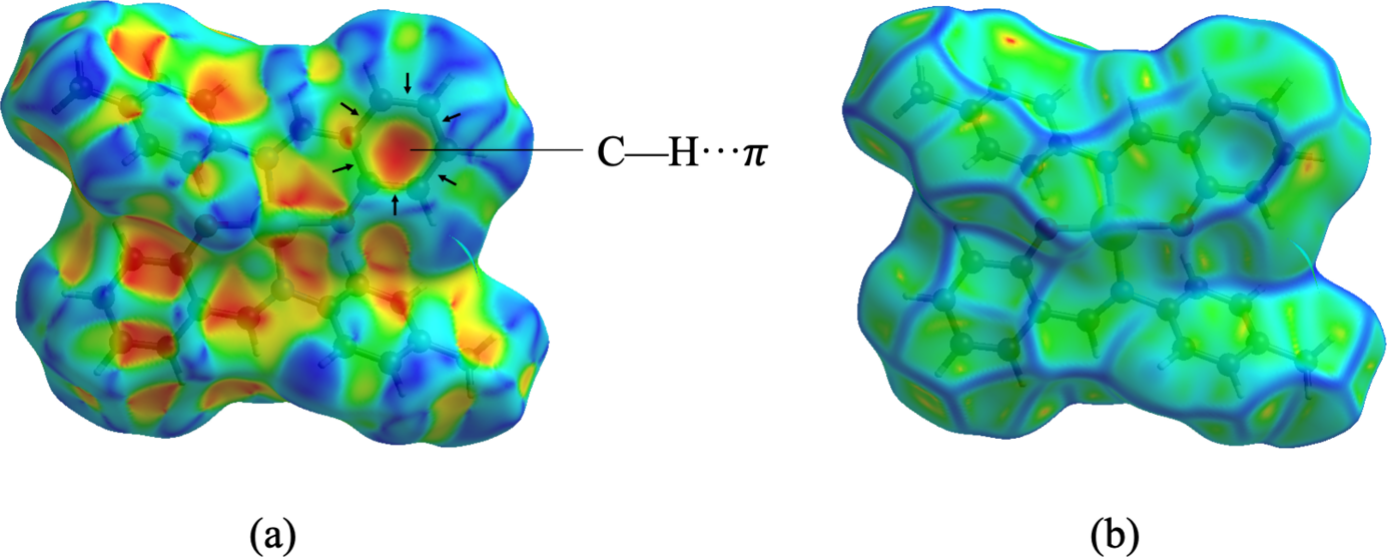
Hirshfeld surface of **1** plotted over (*a*) shape-index and (*b*) curvedness.

In addition, shape-index and curvedness surface analyses were performed to predict the existence of C—H⋯π inter­actions, as shown in Fig. 4 ▸*a* and *b*, respectively. The C—H⋯π inter­action is indicated by the bright-orange concave region marked by black arrows (Luo *et al.*, 2014 ▸) while large flat regions are shown by a blue outline on the curvedness diagram.

**Figure 5:**
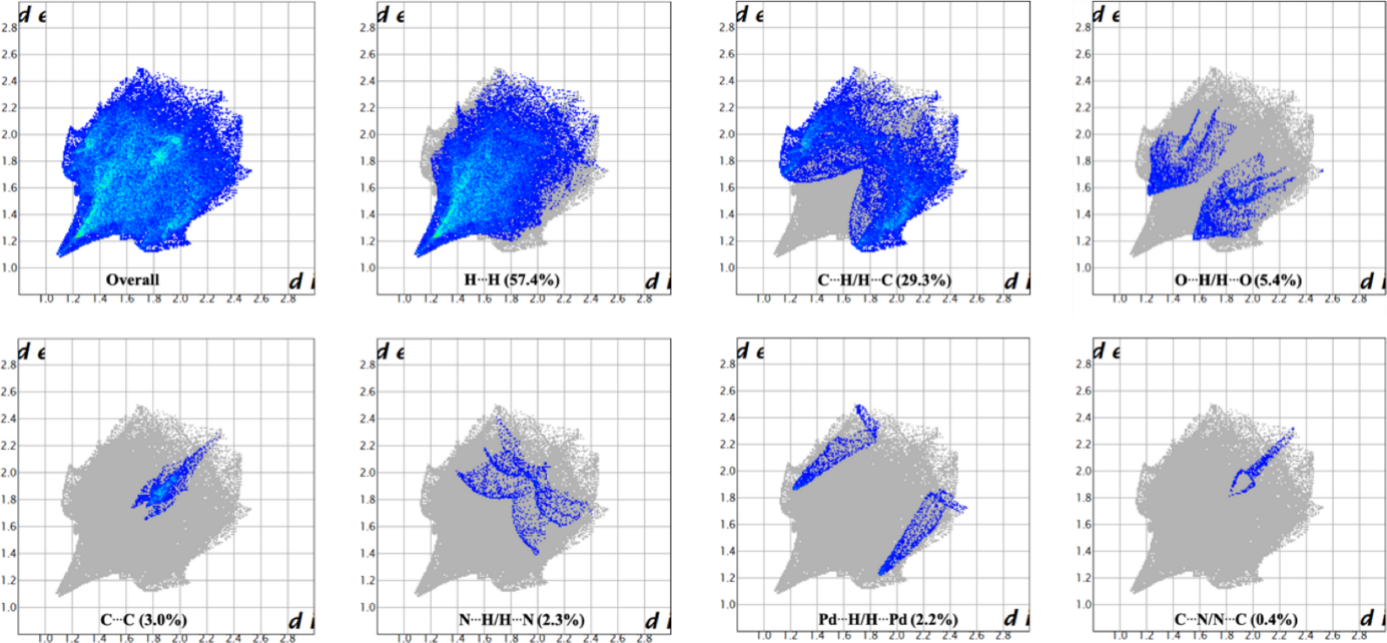
Two-dimensional fingerprint plots for **1**.

The percentage contributions of the inter­molecular inter­actions to the total Hirshfeld surface were qu­anti­fied by two-dimensional fingerprint plots (Suda *et al.*, 2023 ▸). The fingerprint plots of *d*_i_*versus d*_e_ shown in Fig. 5 ▸ reveal that the most significant contributions arise from H⋯H (57.4%) and C⋯H/H⋯C (29.3%) contacts. The wing-like features in the C⋯H/H⋯C plot is another indication of the presence of C—H⋯π inter­actions (Spackman & McKinnon, 2002 ▸). Smaller contributions are observed for O⋯H/H⋯O (5.4%), C⋯C (3.0%), N⋯H/H⋯N (2.3%), Pd⋯H/H⋯Pd (2.2%), and C⋯N/N⋯C (0.4%) inter­actions. Here, the *d*_i_ corresponds to the closest inter­nal distance from a given point on the Hirshfeld surface, while *d*_e_ represents the closest external distance to neighboring mol­ecules.

## 5. Database survey

A search of the Cambridge Structural Database (webCSD accessed October 2025; Groom *et al.*, 2016 ▸) for **1** returned no relevant hits. However, a search with generalized bidentate *N,O*-chelating Schiff base palladium(II) complexes with similar structures returned a number of hits including CSD refcodes COZHAA (Manotti Lanfredi *et al.*, 1985 ▸), GATBOT (Lai *et al.*, 2005 ▸), NENJAR (Zhou *et al.*, 2000 ▸), XEKXUJ (Saxena & Murugavel, 2017 ▸) and XOJHOW (Kassim *et al.*, 2019 ▸). Although JUPWAW (Moreno-Narváez *et al.*, 2025 ▸) features a very similar mol­ecular framework, its packing arrangement differs significantly from **1**. These differences mainly arise from substituent effects, particularly the CF_3_ group, which modifies the inter­molecular contacts and weakens the π–π stacking. A similar behavior is seen in KIKZOX (Waziri *et al.*, 2023 ▸) and XIVGOC (Meena *et al.*, 2023 ▸), where changes in the aromatic rings with different substituents lead to different packing arrangements. This shows that even small substituent changes can significantly affect the overall crystal packing.

## 6. Synthesis and crystallization

The free ligand [CCDC No. 1470130 (Mague & Mohamed, 2016 ▸); 2.113 g, 10 mmol] was dissolved in hot ethanol in a 100 mL round-bottom flask. Palladium(II) acetate (1.123 g, 5 mmol) was dissolved separately in hot ethanol and added into the flask containing the ligand solution. The mixture was stirred and refluxed for 6 h, affording a brown solid. The solid was collected by filtration, washed with ice-cold ethanol, and air-dried at room temperature. Recrystallization by slow evaporation from chloro­form at room temperature yielded orange block crystals of **1**. Yield 92.4%, m.p. 594–595 K. Elemental analysis for C_28_H_24_N_2_O_4_Pd calculated (obtained): C, 63.82 (62.94); H, 4.59 (4.48); N, 5.32 (5.17). UV-Vis (aceto­nitrile, nm) λ_max_, 247 [π–π* (C=C)], 295 [π–π* (C=N)], 416 (*n*–π*), 508 (LMCT). IR (KBr, cm^−1^): 1597 *v*(C=N), 1381 ν(C—N), 1314 ν(C—O), 542 ν(Pd—N), 449 ν(Pd—O). ^1^H NMR (500 MHz, CDCl_3_) δ ppm: 2.45 [*s*, 3H, C^11^—H (Ar)], 6.17–6.20 [*m*, 4H, C^9,10^—H (Ar)], 6.50–6.55 [*m*, 1H, C^4^—H (Ar)], 7.11–7.12 [*m*, 1H, C^5^—H (Ar)], 7.14–7.16 [*m*, 1H, C^3^—H (Ar)], 7.18–7.19 *[m*, 1H, C^2^—H (Ar)], 7.73 (*s*, 1H, HC^7^=N). ^13^C NMR (500 MHz, CDCl_3_) δ ppm: 30.9 (C^12^), 115.1 [C^2^ (Ar)], 120.3 [C^6^ (Ar)], 120.7 [C^4^ (Ar)], 124.4 [C^9^—H (Ar)], 128.6 [C^10^ (Ar)], 134.4 [C^5^ (Ar)], 135.0 [C^3^ (Ar)], 136.1 [C^11^ (Ar)], 147.1 [C^8^ (Ar)], 162.7 (C^7^=N), 165.2 (C^1^).

## 7. Refinement

Crystal data, data collection and structure refinement details are summarized in Table 3 ▸. H atoms were positioned geometrically (0.93–0.96 Å) and refined as riding with *U*_iso_(H) = 1.2–1.5*U*_eq_(C).

## Supplementary Material

Crystal structure: contains datablock(s) I. DOI: 10.1107/S2056989025011570/oo2015sup1.cif

Structure factors: contains datablock(s) I. DOI: 10.1107/S2056989025011570/oo2015Isup2.hkl

1H NMR spectrum. DOI: 10.1107/S2056989025011570/oo2015sup3.png

13C NMR spectrum. DOI: 10.1107/S2056989025011570/oo2015sup4.png

IR spectrum. DOI: 10.1107/S2056989025011570/oo2015sup5.png

UV-Vis spectrum. DOI: 10.1107/S2056989025011570/oo2015sup6.png

CCDC reference: 2286186

Additional supporting information:  crystallographic information; 3D view; checkCIF report

## Figures and Tables

**Table 1:** Selected bond lengths (Å) and bond angles (°) in **1**

Bond lengths (Å)		Bond angles (°)	
Pd1—O1	1.969 (3)	Pd1—O1—C1	125.4 (2)
O1—C1	1.316 (5)	N2—Pd1—O1	91.41 (11)
Pd1—N2	2.023 (3)	Pd1—N2—C7	123.4 (2)
N2—C7	1.290 (4)	Pd1—N2—C8	120.0 (2)
N2—C8	1.443 (5)		

**Table 2:** C—H⋯π inter­action (Å, °)

*D*—H⋯*A*	*D*—H, π	H⋯*A*	*D*⋯*A*	*D*—H⋯*A*
C11—H11⋯*Cg*3^i^	34	2.81	3.542 (5)	136

Symmetry code: (i) *x*, 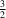 − *y*, 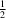 + *z*. *Cg*3 is the centroid of the C1–C6 ring.

**Table 3:** Experimental details

Crystal data	
Chemical formula	[Pd(C_14_H_12_NO)_2_]
*M*_r_	526.89
Crystal system, space group	Monoclinic, *P*2_1_/*c*
Temperature (K)	299
*a*, *b*, *c* (Å)	9.941 (3), 10.952 (3), 10.969 (3)
β (°)	105.337 (8)
*V* (Å^3^)	1151.8 (5)
*Z*	2
Radiation type	Mo *K*α
μ (mm^−1^)	0.83
Crystal size (mm)	0.27 × 0.23 × 0.17

Data collection	
Diffractometer	Bruker APEXII CCD
Absorption correction	Multi-scan (*SADABS*; Krause *et al.*, 2015 ▸)
*T*_min_, *T*_max_	0.640, 0.746
No. of measured, independent and observed [*I* > 2σ(*I*)] reflections	28335, 2866, 2202
*R*_int_	0.041
(sin θ/λ)_max_ (Å^−1^)	0.667

Refinement	
*R*[*F*^2^ > 2σ(*F*^2^)], *wR*(*F*^2^), *S*	0.043, 0.102, 1.26
No. of reflections	2866
No. of parameters	152
H-atom treatment	H-atom parameters constrained
Δρ_max_, Δρ_min_ (e Å^−3^)	0.86, −0.59

Computer programs: *APEX2* and *SAINT* (Bruker, 2014 ▸), *SHELXTL* (Sheldrick, 2015*a* ▸), *SHELXL2014/7* (Sheldrick, 2015*b* ▸) and *OLEX2* (Dolomanov *et al.*, 2009 ▸).

## supplementary crystallographic information

### Bis{2-[(E)-(p-tolylimino)methyl]benzen-1-olato}palladium Crystal data

**Table:**

[Pd(C_14_H_12_NO)_2_]	*F*(000) = 536
*M**_r_* = 526.89	*D*_x_ = 1.519 Mg m^−^^3^
Monoclinic, *P*2_1_/*c*	Mo *K*α radiation, λ = 0.71073 Å
*a* = 9.941 (3) Å	Cell parameters from 8213 reflections
*b* = 10.952 (3) Å	θ = 2.7–28.3°
*c* = 10.969 (3) Å	µ = 0.83 mm^−^^1^
β = 105.337 (8)°	*T* = 299 K
*V* = 1151.8 (5) Å^3^	Block, orange
*Z* = 2	0.27 × 0.23 × 0.17 mm

### Bis{2-[(E)-(p-tolylimino)methyl]benzen-1-olato}palladium Data collection

**Table:**

Bruker APEXII CCD diffractometer	2202 reflections with *I* > 2σ(*I*)
φ and ω scans	*R*_int_ = 0.041
Absorption correction: multi-scan (SADABS; Krause *et al.*, 2015)	θ_max_ = 28.3°, θ_min_ = 3.7°
*T*_min_ = 0.640, *T*_max_ = 0.746	*h* = −13→13
28335 measured reflections	*k* = −14→14
2866 independent reflections	*l* = −14→14

### Bis{2-[(E)-(p-tolylimino)methyl]benzen-1-olato}palladium Refinement

**Table:**

Refinement on *F*^2^	0 restraints
Least-squares matrix: full	Hydrogen site location: inferred from neighbouring sites
*R*[*F*^2^ > 2σ(*F*^2^)] = 0.043	H-atom parameters constrained
*wR*(*F*^2^) = 0.102	*w* = 1/[σ^2^(*F*_o_^2^) + 2.4894*P*] where *P* = (*F*_o_^2^ + 2*F*_c_^2^)/3
*S* = 1.26	(Δ/σ)_max_ < 0.001
2866 reflections	Δρ_max_ = 0.86 e Å^−^^3^
152 parameters	Δρ_min_ = −0.59 e Å^−^^3^

### Bis{2-[(E)-(p-tolylimino)methyl]benzen-1-olato}palladium Special details

**Table:**

Geometry. All esds (except the esd in the dihedral angle between two l.s. planes) are estimated using the full covariance matrix. The cell esds are taken into account individually in the estimation of esds in distances, angles and torsion angles; correlations between esds in cell parameters are only used when they are defined by crystal symmetry. An approximate (isotropic) treatment of cell esds is used for estimating esds involving l.s. planes.

### Bis{2-[(E)-(p-tolylimino)methyl]benzen-1-olato}palladium Fractional atomic coordinates and isotropic or equivalent isotropic displacement parameters (Å^2^)

**Table:**

	*x*	*y*	*z*	*U*_iso_*/*U*_eq_	
Pd1	0.500000	0.500000	0.500000	0.03766 (12)	
O1	0.3935 (3)	0.4667 (3)	0.3246 (2)	0.0581 (7)	
N2	0.5448 (3)	0.6713 (2)	0.4539 (3)	0.0392 (6)	
C1	0.3344 (4)	0.5497 (3)	0.2408 (3)	0.0428 (7)	
C2	0.2337 (4)	0.5091 (4)	0.1321 (4)	0.0515 (9)	
H2	0.208633	0.427112	0.123439	0.062*	
C3	0.1729 (5)	0.5909 (5)	0.0392 (4)	0.0630 (12)	
H3	0.104590	0.563363	−0.030722	0.076*	
C4	0.2103 (5)	0.7145 (5)	0.0461 (4)	0.0646 (12)	
H4	0.168698	0.768064	−0.018704	0.078*	
C5	0.3086 (4)	0.7546 (4)	0.1495 (4)	0.0544 (9)	
H5	0.334922	0.836317	0.154478	0.065*	
C6	0.3715 (4)	0.6750 (3)	0.2496 (3)	0.0413 (7)	
C7	0.4794 (4)	0.7252 (3)	0.3506 (3)	0.0415 (7)	
H7	0.505099	0.805438	0.340683	0.050*	
C8	0.6467 (4)	0.7435 (3)	0.5430 (3)	0.0385 (7)	
C9	0.7833 (4)	0.7047 (4)	0.5833 (4)	0.0537 (9)	
H9	0.810674	0.632149	0.552762	0.064*	
C10	0.8788 (4)	0.7742 (4)	0.6691 (4)	0.0570 (10)	
H10	0.971174	0.748409	0.694419	0.068*	
C11	0.6069 (4)	0.8514 (3)	0.5879 (3)	0.0432 (8)	
H11	0.515493	0.878994	0.559108	0.052*	
C12	0.7042 (4)	0.9185 (3)	0.6764 (4)	0.0515 (9)	
H12	0.676592	0.990310	0.708025	0.062*	
C13	0.8418 (4)	0.8808 (3)	0.7188 (4)	0.0493 (9)	
C14	0.9460 (5)	0.9557 (5)	0.8140 (5)	0.0717 (13)	
H14A	0.908489	1.035761	0.819350	0.108*	
H14B	1.030731	0.962422	0.788258	0.108*	
H14C	0.965089	0.916874	0.895262	0.108*	

### Bis{2-[(E)-(p-tolylimino)methyl]benzen-1-olato}palladium Atomic displacement parameters (Å^2^)

**Table:**

	*U*^11^	*U*^22^	*U*^33^	*U*^12^	*U*^13^	*U*^23^
Pd1	0.0499 (2)	0.02614 (17)	0.03647 (19)	−0.00358 (14)	0.01066 (15)	−0.00313 (13)
O1	0.081 (2)	0.0433 (14)	0.0403 (14)	−0.0127 (14)	−0.0009 (13)	−0.0049 (11)
N2	0.0454 (15)	0.0315 (13)	0.0402 (14)	−0.0016 (11)	0.0102 (12)	−0.0040 (11)
C1	0.0488 (19)	0.0414 (18)	0.0391 (17)	−0.0018 (15)	0.0132 (15)	−0.0034 (14)
C2	0.053 (2)	0.058 (2)	0.0423 (19)	−0.0057 (18)	0.0097 (16)	−0.0121 (17)
C3	0.054 (2)	0.087 (3)	0.042 (2)	0.003 (2)	0.0022 (18)	−0.013 (2)
C4	0.067 (3)	0.067 (3)	0.054 (2)	0.017 (2)	0.005 (2)	0.005 (2)
C5	0.061 (2)	0.049 (2)	0.051 (2)	0.0110 (18)	0.0102 (18)	0.0020 (17)
C6	0.0460 (18)	0.0410 (18)	0.0377 (17)	0.0018 (14)	0.0123 (14)	−0.0023 (14)
C7	0.0488 (19)	0.0320 (16)	0.0446 (18)	−0.0010 (14)	0.0139 (15)	−0.0008 (14)
C8	0.0431 (17)	0.0318 (15)	0.0405 (17)	−0.0058 (13)	0.0108 (14)	−0.0006 (13)
C9	0.049 (2)	0.0386 (19)	0.070 (3)	0.0025 (16)	0.0101 (19)	−0.0075 (18)
C10	0.045 (2)	0.051 (2)	0.068 (3)	−0.0005 (17)	0.0040 (19)	0.0013 (19)
C11	0.0460 (19)	0.0380 (17)	0.0467 (19)	−0.0040 (14)	0.0142 (15)	−0.0061 (14)
C12	0.061 (2)	0.0366 (18)	0.057 (2)	−0.0044 (17)	0.0158 (19)	−0.0122 (16)
C13	0.055 (2)	0.0422 (19)	0.047 (2)	−0.0107 (16)	0.0058 (17)	−0.0014 (16)
C14	0.071 (3)	0.067 (3)	0.065 (3)	−0.020 (2)	−0.003 (2)	−0.010 (2)

### Bis{2-[(E)-(p-tolylimino)methyl]benzen-1-olato}palladium Geometric parameters (Å, º)

**Table:**

Pd1—O1	1.969 (3)	C6—C7	1.432 (5)
Pd1—O1^i^	1.969 (3)	C7—H7	0.9300
Pd1—N2^i^	2.023 (3)	C8—C9	1.380 (5)
Pd1—N2	2.023 (3)	C8—C11	1.378 (5)
O1—C1	1.316 (5)	C9—H9	0.9300
N2—C7	1.290 (4)	C9—C10	1.376 (6)
N2—C8	1.443 (4)	C10—H10	0.9300
C1—C2	1.411 (5)	C10—C13	1.379 (6)
C1—C6	1.418 (5)	C11—H11	0.9300
C2—H2	0.9300	C11—C12	1.386 (5)
C2—C3	1.371 (6)	C12—H12	0.9300
C3—H3	0.9300	C12—C13	1.386 (6)
C3—C4	1.401 (7)	C13—C14	1.505 (5)
C4—H4	0.9300	C14—H14A	0.9600
C4—C5	1.359 (6)	C14—H14B	0.9600
C5—H5	0.9300	C14—H14C	0.9600
C5—C6	1.412 (5)		
			
O1—Pd1—O1^i^	180.0	N2—C7—C6	127.2 (3)
O1^i^—Pd1—N2	88.59 (11)	N2—C7—H7	116.4
O1—Pd1—N2	91.41 (11)	C6—C7—H7	116.4
O1—Pd1—N2^i^	88.59 (11)	C9—C8—N2	120.2 (3)
O1^i^—Pd1—N2^i^	91.41 (11)	C11—C8—N2	119.7 (3)
N2^i^—Pd1—N2	180.0	C11—C8—C9	120.1 (3)
C1—O1—Pd1	125.4 (2)	C8—C9—H9	120.3
C7—N2—Pd1	123.4 (2)	C10—C9—C8	119.4 (4)
C7—N2—C8	116.4 (3)	C10—C9—H9	120.3
C8—N2—Pd1	120.0 (2)	C9—C10—H10	119.0
O1—C1—C2	117.3 (3)	C9—C10—C13	122.1 (4)
O1—C1—C6	124.1 (3)	C13—C10—H10	119.0
C2—C1—C6	118.4 (3)	C8—C11—H11	120.3
C1—C2—H2	120.1	C8—C11—C12	119.5 (4)
C3—C2—C1	119.8 (4)	C12—C11—H11	120.3
C3—C2—H2	120.1	C11—C12—H12	119.3
C2—C3—H3	118.9	C11—C12—C13	121.4 (3)
C2—C3—C4	122.2 (4)	C13—C12—H12	119.3
C4—C3—H3	118.9	C10—C13—C12	117.5 (3)
C3—C4—H4	120.7	C10—C13—C14	122.0 (4)
C5—C4—C3	118.6 (4)	C12—C13—C14	120.5 (4)
C5—C4—H4	120.7	C13—C14—H14A	109.5
C4—C5—H5	119.3	C13—C14—H14B	109.5
C4—C5—C6	121.5 (4)	C13—C14—H14C	109.5
C6—C5—H5	119.3	H14A—C14—H14B	109.5
C1—C6—C7	123.5 (3)	H14A—C14—H14C	109.5
C5—C6—C1	119.4 (3)	H14B—C14—H14C	109.5
C5—C6—C7	116.9 (3)		
			
Pd1—O1—C1—C2	−164.1 (3)	C4—C5—C6—C1	1.8 (6)
Pd1—O1—C1—C6	19.8 (5)	C4—C5—C6—C7	176.6 (4)
Pd1—N2—C7—C6	−3.2 (5)	C5—C6—C7—N2	175.7 (4)
Pd1—N2—C8—C9	61.7 (4)	C6—C1—C2—C3	−1.0 (6)
Pd1—N2—C8—C11	−118.1 (3)	C7—N2—C8—C9	−123.2 (4)
O1—C1—C2—C3	−177.4 (4)	C7—N2—C8—C11	56.9 (4)
O1—C1—C6—C5	175.3 (4)	C8—N2—C7—C6	−178.1 (3)
O1—C1—C6—C7	0.8 (6)	C8—C9—C10—C13	1.5 (7)
N2—C8—C9—C10	−179.5 (4)	C8—C11—C12—C13	1.5 (6)
N2—C8—C11—C12	178.0 (3)	C9—C8—C11—C12	−1.8 (5)
C1—C2—C3—C4	2.0 (7)	C9—C10—C13—C12	−1.8 (6)
C1—C6—C7—N2	−9.7 (6)	C9—C10—C13—C14	179.1 (4)
C2—C1—C6—C5	−0.8 (5)	C11—C8—C9—C10	0.4 (6)
C2—C1—C6—C7	−175.3 (3)	C11—C12—C13—C10	0.3 (6)
C2—C3—C4—C5	−1.1 (7)	C11—C12—C13—C14	179.4 (4)
C3—C4—C5—C6	−0.8 (7)		

Symmetry code: (i) −*x*+1, −*y*+1, −*z*+1.
