# Supplementary figures and images for: Synthesis, crystal structure, and Hirshfeld surface analysis of bis­{2-[(E)-(p-tolyl­imino)­meth­yl]benzen-1-olato}palladium
Source: Acta Crystallogr E Crystallogr Commun. 2026 Jan 6;82(Pt 2):138–42. doi: 10.1107/S2056989025011570 (PMC12874235; doi:10.1107/S2056989025011570)

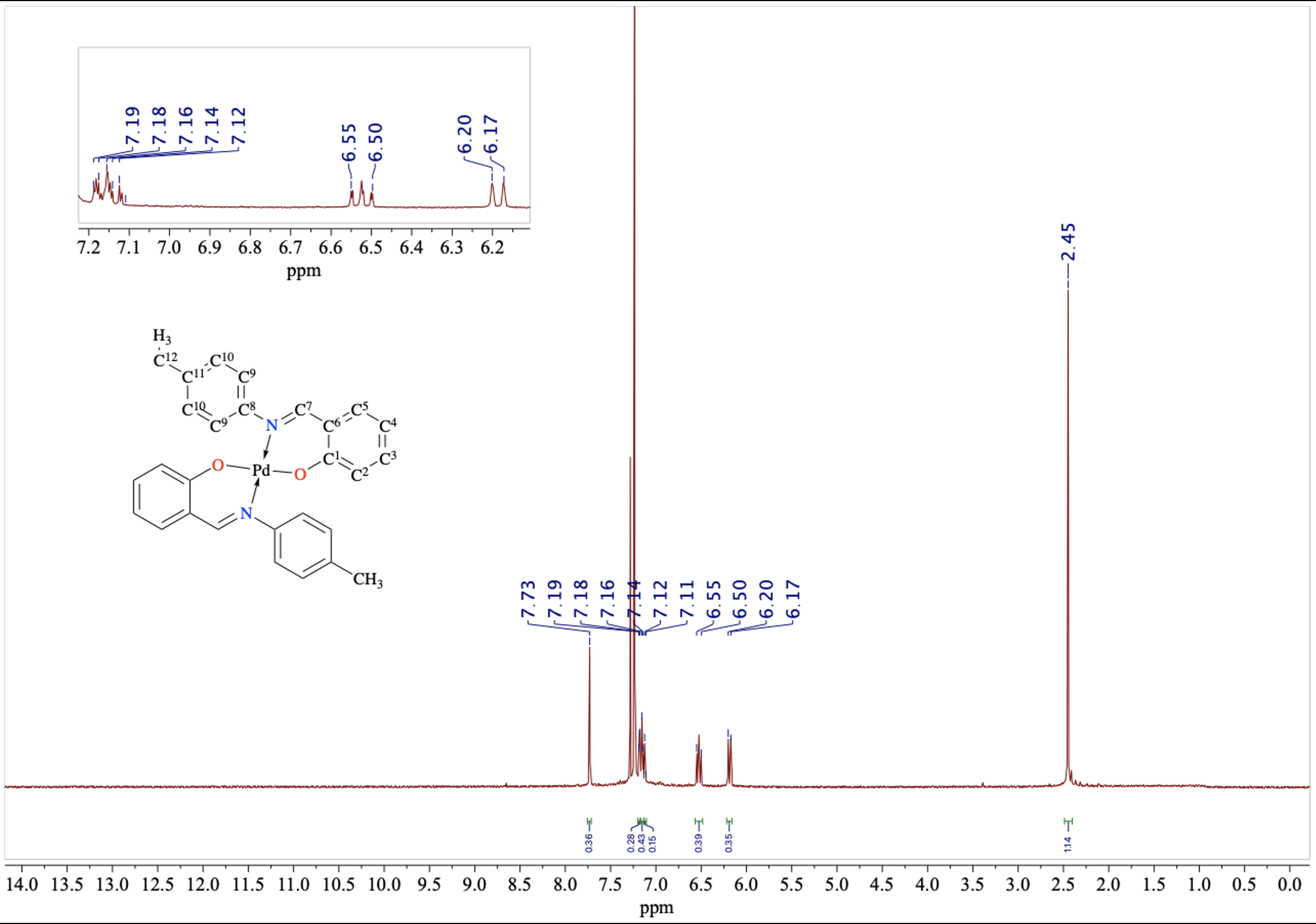

Supplement: Supplementary file 3 [file e-82-00138-sup3.png]

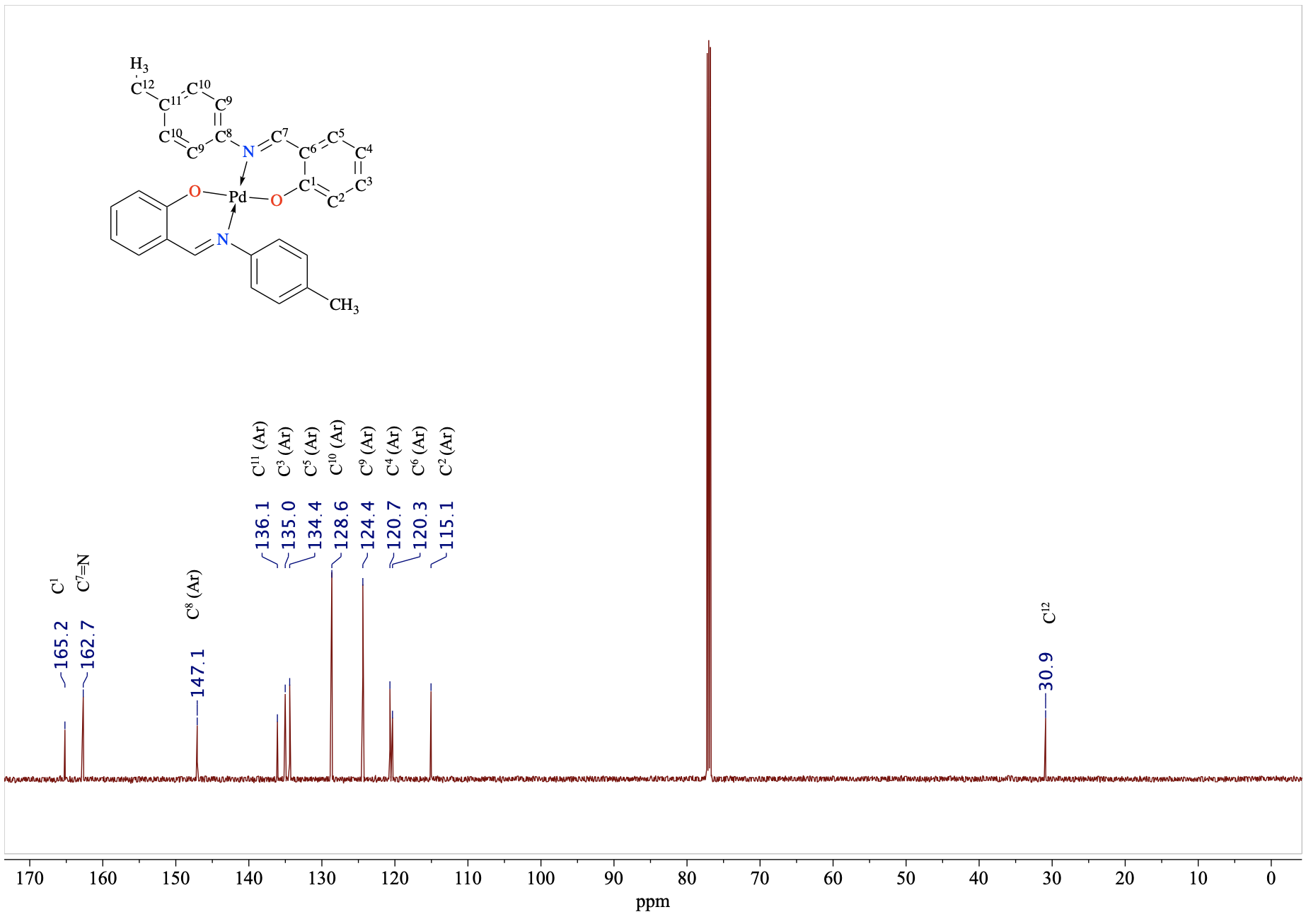

Supplement: Supplementary file 4 [file e-82-00138-sup4.png]

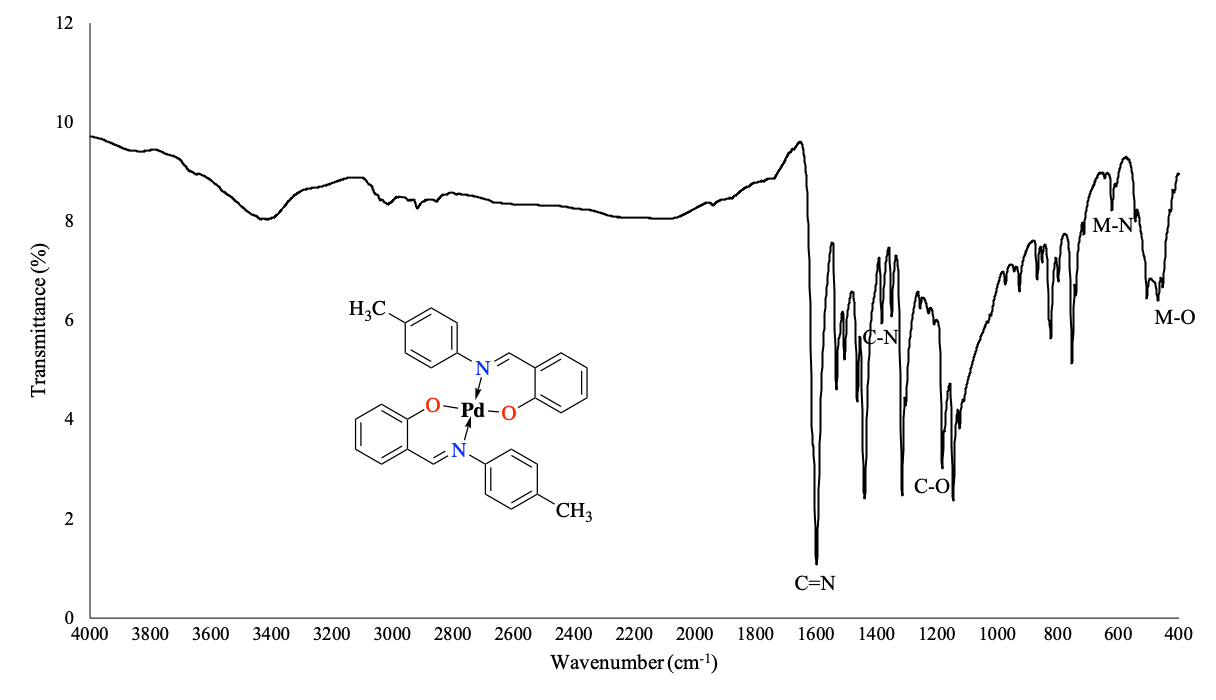

Supplement: Supplementary file 5 [file e-82-00138-sup5.png]

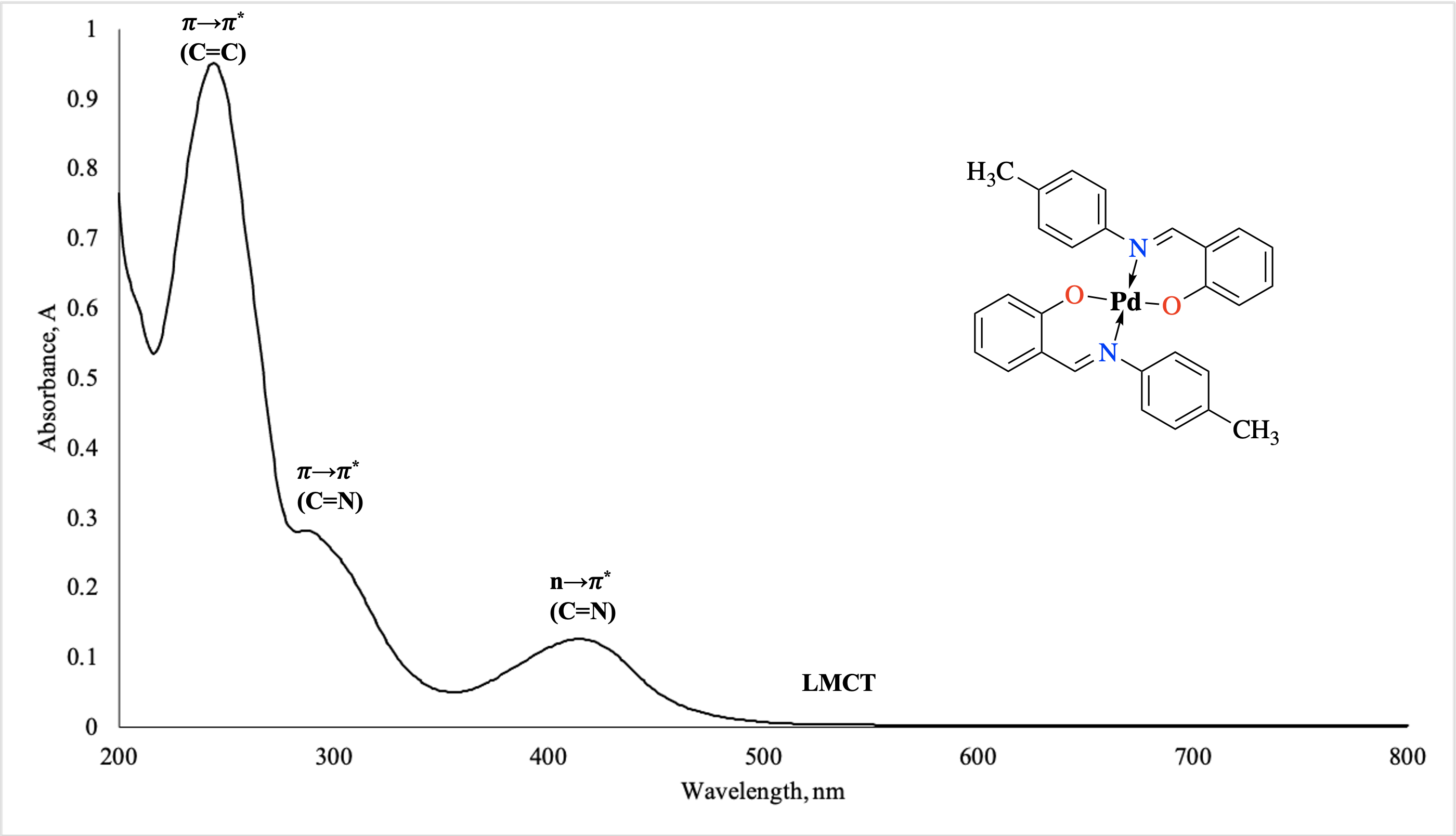

Supplement: Supplementary file 6 [file e-82-00138-sup6.png]
